# Intestinal Ultrasound of Adult Small Bowel Intussusception Caused by a Polyp: A Case Report

**DOI:** 10.1002/ccr3.72637

**Published:** 2026-04-28

**Authors:** Oleksii Dutchak, Maksym Zhaivoronok

**Affiliations:** ^1^ “Ultramed” Medical Center LLC Lviv Ukraine; ^2^ Department of Nuclear Medicine Radiation Oncology and Radiation Safety of Shupyk National Healthcare University of Ukraine Kyiv Ukraine

**Keywords:** intestinal polyp, intestinal ultrasound, intussusception, small bowel, ultrasoundcase report

## Abstract

Intestinal ultrasound may facilitate early diagnosis of adult small bowel intussusception and can directly demonstrate a pathological lead point such as an intraluminal polyp. In patients with nonspecific abdominal symptoms, this may expedite CT confirmation, support appropriate surgical referral, and improve diagnostic confidence and overall clinical decision‐making and management.

## Graphical abstract

Intestinal ultrasound findings in adult small bowel intussusception caused by a polyp.

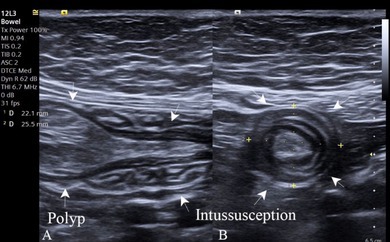

## 1 Introduction

Intussusception refers to the telescoping of one segment of the gastrointestinal tract into an adjacent segment, leading to bowel obstruction and potential ischemia [1]. While intussusception is a common cause of acute abdomen in children, it accounts for only approximately 5% of all cases in adults [2]. In contrast to pediatric intussusception, which is often idiopathic, adult intussusception is typically secondary to an identifiable pathological lead point, including benign or malignant tumors, polyps, Meckel's diverticulum, or postoperative changes [3].

Computed tomography (СТ) is widely regarded as the reference imaging modality for diagnosing adult intussusception [4]. At the same time, ultrasound examination, particularly intestinal ultrasound (IUS), remains a valuable, widely available, and radiation‐free imaging modality, especially in the initial assessment of patients presenting with abdominal pain [5]. Despite this, ultrasound‐based descriptions, including those using IUS, of adult small bowel intussusception with direct visualization of the causative lead point remain relatively scarce in the literature. Accordingly, this report focuses on the preoperative sonographic identification of the structural lead point, with subsequent CT, surgical, and histopathological confirmation.

## 2 Case History/Examination

A 36‐year‐old woman presented with intermittent, cramp‐like abdominal pain lasting 3 days. The pain was predominantly localized to the periumbilical region and was occasionally associated with nausea, without vomiting or gastrointestinal bleeding. On physical examination, mild abdominal tenderness was noted, with no signs of peritoneal irritation. Laboratory investigations, including complete blood count and inflammatory markers, were within normal limits.

**FIGURE 1:**
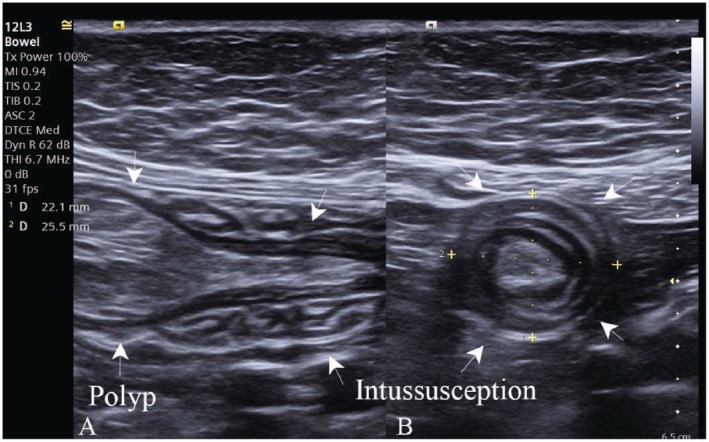
IUS findings in adult small bowel intussusception caused by an intraluminal polyp. (A) Longitudinal IUS image demonstrating an intussuscepted segment of the small bowel with a well‐circumscribed intraluminal polypoid lesion (arrows), considered the likely causative lead point. (B) Transverse IUS image showing a characteristic concentric multilayered configuration consistent with small bowel intussusception (“target” or “donut” sign).

**FIGURE 2:**
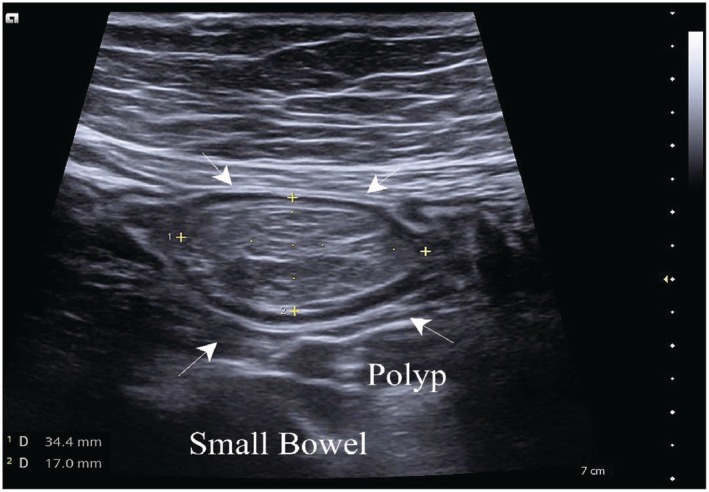
Longitudinal IUS image of the small bowel with an intraluminal polyp. A well‐circumscribed intraluminal polypoid lesion (arrows) measuring 34 × 17 mm is visualized and was considered the likely causative lead point.

Abdominal ultrasound was performed using a high‐frequency linear transducer (3–12 MHz), complemented by a low‐frequency convex probe for deeper assessment. Examination revealed a characteristic concentric, multilayered appearance of the bowel in the transverse plane (“target” or “donut” sign), consistent with intussusception. In the longitudinal plane, a reniform configuration (“pseudokidney sign”) was observed (Figure 1). Within the intussuscepted segment, a well‐circumscribed, oval‐shaped intraluminal polypoid lesion measuring approximately 34 × 17 mm was identified and was considered a likely causative lead point (Figure 2). Color Doppler imaging demonstrated preserved vascularity of the involved bowel walls, with no sonographic signs of bowel ischemia or perforation. Reduced peristalsis was observed in the affected bowel segment.

## 3 Differential Diagnosis, Investigations and Treatment

Based on the clinical presentation and IUS findings, persistent small bowel intussusception caused by a structural lead point was considered the most likely diagnosis. This interpretation was supported by the presence of ongoing symptoms, reduced peristalsis in the affected bowel segment, and direct visualization of a well‐defined intraluminal polypoid lesion within the intussuscepted segment.

**FIGURE 3:**
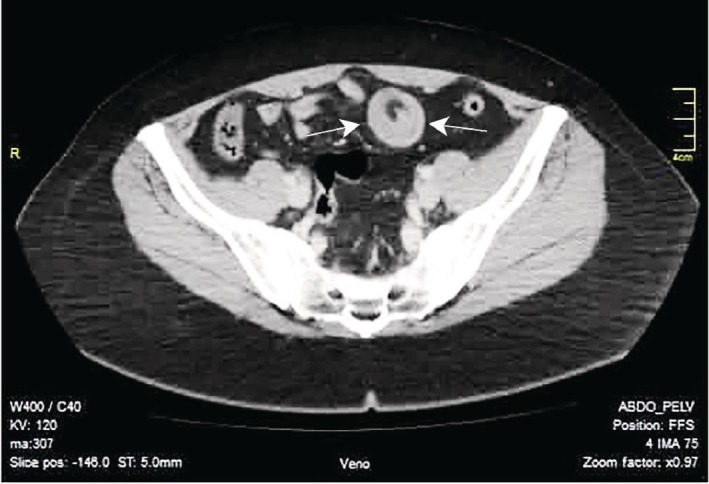
Axial contrast‐enhanced CT image demonstrating small bowel intussusception with a characteristic bowel‐within‐bowel configuration (arrows) and an intraluminal lead point, without signs of ischemia or perforation.

To further evaluate the IUS findings and confirm the diagnosis, contrast‐enhanced CT of the abdomen was performed. CT imaging confirmed persistent small bowel intussusception and identified an intraluminal lesion consistent with a polypoid lead point. No signs of bowel wall ischemia, perforation, or free intraperitoneal air were observed. The CT findings were concordant with the ultrasound assessment (Figure 3).

Operative management was selected on the basis of both the clinical presentation and the imaging findings. Specifically, the patient had persistent symptoms (intermittent cramp‐like abdominal pain and nausea), while both IUS and contrast‐enhanced CT demonstrated persistent small bowel intussusception with a clearly identifiable structural lead point. In this clinical and imaging context, surgical exploration was considered the most appropriate management strategy. Intraoperative exploration confirmed small bowel intussusception caused by an intraluminal polyp. Segmental resection of the affected bowel was performed.

## 4 Conclusion and Results (Outcome and Follow‐Up)

**FIGURE 4:**
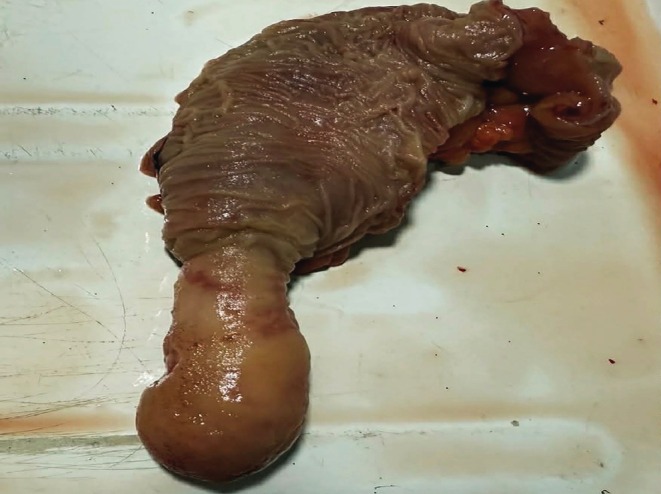
Gross pathological specimen following surgical resection. The resected segment of the small bowel demonstrates an intraluminal polypoid lesion corresponding to the causative lead point of intussusception. The macroscopic appearance is consistent with a benign polyp, later confirmed by histopathological examination.

Histopathological analysis of the resected specimen revealed a benign intestinal adenomatous polyp, with no evidence of malignancy (Figure 4). The postoperative course was uneventful, and the patient was discharged in good clinical condition. No early postoperative complications were reported. Thus, the final diagnosis of persistent adult small bowel intussusception caused by a benign polypoid lead point was confirmed by combined imaging, surgery, and histopathology.

This case underscores the role of ultrasound as an effective first‐line imaging modality for adult small bowel intussusception. Importantly, ultrasound may allow direct identification of the causative lead point, such as an intestinal polyp, thereby facilitating timely confirmatory imaging, surgical referral, and definitive management.

## 5 Discussion

Adult small bowel intussusception is a rare but clinically significant condition, often associated with an underlying structural abnormality [6]. Identification of a lead point is clinically important, as in adult patients it often supports the need for surgical intervention [7]. In the present case, the most notable finding was not only the sonographic detection of intussusception itself, but also the direct visualization of an intraluminal polypoid lesion acting as the lead point [8]. This represents the principal diagnostic strength of the present case and confers practical relevance, as preoperative sonographic visualization of the lead point remains relatively uncommon in the published literature, particularly in cases with clear imaging–surgical–histopathological correlation. Direct sonographic identification of the lead point is of particular clinical relevance in adults, as it increases diagnostic confidence and may facilitate timely referral for definitive management [9].

CT findings were fully concordant with the ultrasound assessment, confirming the diagnosis, verifying the intraluminal lead point, and showing no evidence of perforation or ischemic complications. In this setting, ultrasound served as the first‐line imaging modality and facilitated timely confirmatory cross‐sectional imaging and treatment planning. Rather than replacing CT, ultrasound complemented it by providing an early bedside diagnosis and raising suspicion of a structural lead point prior to surgery.

Surgical management remains the standard approach in most adult cases because of the high likelihood of an underlying pathological lead point [10]. In the present patient, operative treatment was selected on the basis of both the clinical presentation and imaging findings indicating persistent small bowel intussusception with a clearly identifiable lead point. Histopathological examination revealed a benign intestinal polyp, in keeping with previous reports showing that adult small bowel intussusception is more often associated with benign lesions. A recent similar case reported by Valeriani et al. likewise emphasized the importance of surgical treatment in adult small bowel intussusception caused by a focal benign lesion [11].

Overall, the relevance of the present case is primarily practical rather than conceptual. Its main added value lies in the preoperative sonographic identification of the structural lead point, with subsequent CT, surgical, and histopathological confirmation. The key take‐home message is that systematic intestinal ultrasound may provide actionable diagnostic information in selected adult patients with nonspecific abdominal symptoms by suggesting both the presence of persistent pathological intussusception and its likely underlying cause. In this context, intestinal ultrasound may improve early triage and support timely referral for definitive management, even though CT remains the reference cross‐sectional imaging modality.

Limitations of ultrasound include operator dependency, bowel gas interference, and reduced image quality in obese patients. Nevertheless, in experienced hands, ultrasound can provide essential diagnostic information even in adult patients.

## Author Contributions

**Oleksii Dutchak:** data curation, resources, validation, visualization, writing – review and editing. **Maksym Zhaivoronok:** conceptualization, formal analysis, methodology, project administration, software, supervision, writing – original draft.

## Funding

The authors have nothing to report.

## Consent

Written informed consent was obtained from the patient for publication of this case report and accompanying images.

## Conflicts of Interest

The authors declare no conflicts of interest.

## Data Availability

Data sharing is not applicable to this article as no datasets were generated or analysed beyond the clinical information presented in this case report.
